# Evolving DNA motifs to predict GeneChip probe performance

**DOI:** 10.1186/1748-7188-4-6

**Published:** 2009-03-19

**Authors:** WB Langdon, AP Harrison

**Affiliations:** 1 Department of Computer Science, King's College London, Strand, London, WC2R 2LS, UK; 2 Biological Sciences, University of Essex, Wivenhoe Park, Colchester, CO4 3SQ, UK

## Abstract

**Background:**

Affymetrix High Density Oligonuclotide Arrays (HDONA) simultaneously measure expression of thousands of genes using millions of probes. We use correlations between measurements for the same gene across 6685 human tissue samples from NCBI's GEO database to indicated the quality of individual HG-U133A probes. Low correlation indicates a poor probe.

**Results:**

Regular expressions can be automatically created from a Backus-Naur form (BNF) context-free grammar using strongly typed genetic programming.

**Conclusion:**

The automatically produced motif is better at predicting poor DNA sequences than an existing human generated RE, suggesting runs of Cytosine and Guanine and mixtures should all be avoided.

## Background

**Figure 1:**
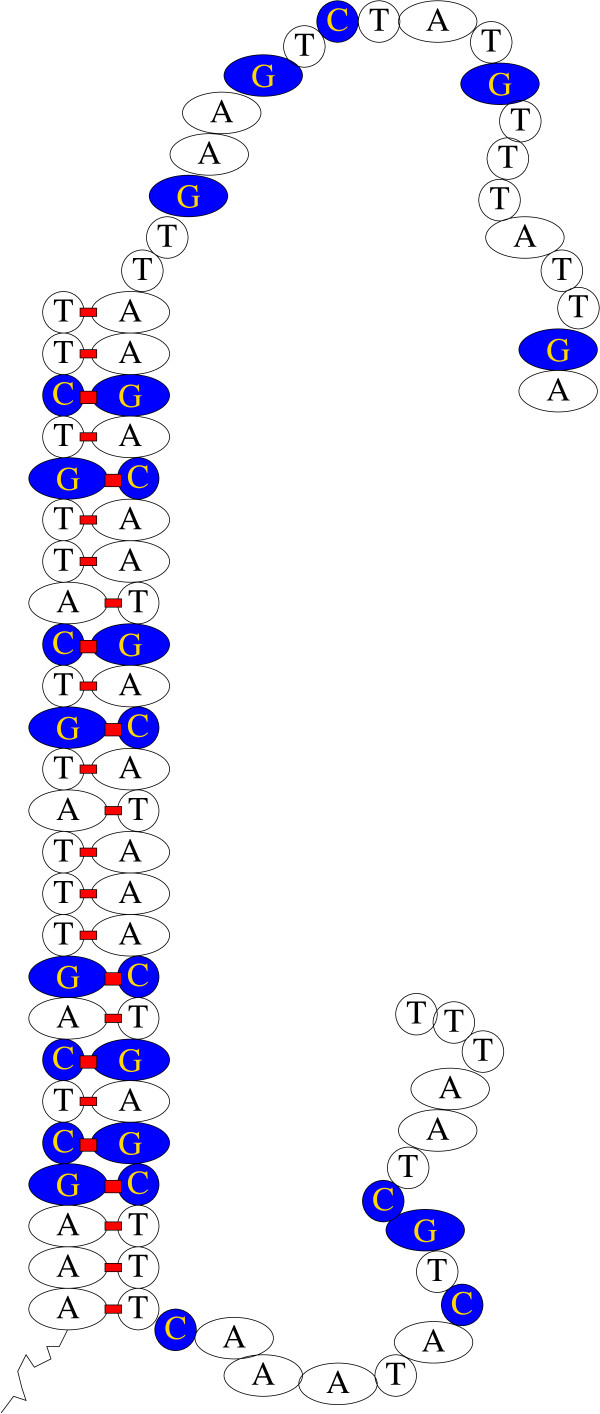
**Schematic of an Affymetrix probe (209649_at PM_5_, left) bound with complementary target sequence (right)**. DNA double helix represented as straight vertical ladder. Note complementary T-A and C-G base bindings are shown by red rectangles. The 25 bases of the probe are tethered to the slide by a flexible linker (black lower left). Firmly bound target sequences can be detected by treatment with a florescent dye, whose location is detected with a laser and an optical microscope. The florescent intensity is approximately proportional to the amount of bound target and so gives some indication of target gene activity.

Typically Affymetrix GeneChips (e.g. HG-U133A) measure gene expression at least eleven points along the gene. Individual measurements are given by short (25 base) DNA sequences, known as probes. These are complementary to corresponding locations in genes. Being complementary, the gene product (messenger RNA) preferentially binds to the probe, cf. Figure 1. Half a million different probes are placed on a slide in a square grid pattern. A fluorescent dye is used to measure how much mRNA is bound to each probe.

**Figure 2:**
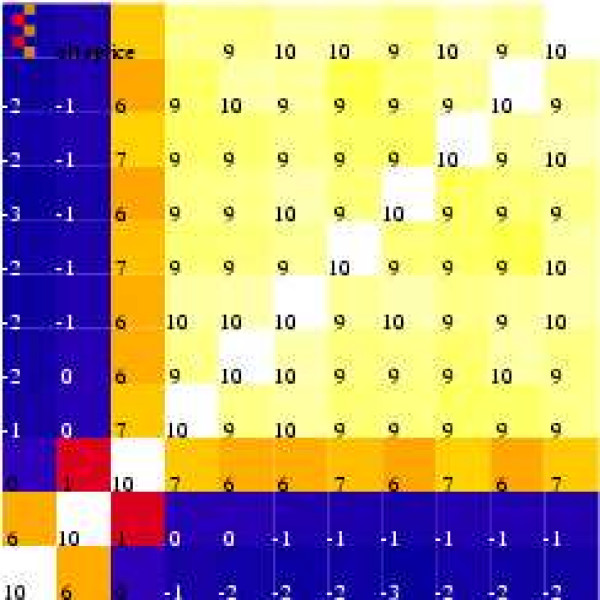
**Correlation coefficients (×10) between 11 probes for gene "S100 calcium binding protein A11" S100A11**. Nine of the probes are correlated but PM_1 _and PM_2 _(bottom 2 rows and 2 left) are not.

To a first approximation, the amount of mRNA produced by a gene should be the same no matter which part of the mRNA molecule is bound to a probe. Affymetrix groups probes into probesets. Each probeset targets a gene. Therefore probe measurements for the same probeset should be correlated. Figure 2 shows the 110 correlations for a probeset as a "heatmap" (yellow/lighter corresponds to greater consistency between pairs of probes). Figure 2 suggests that in Affymetrix probeset 200660_at two probes do *not *measure the gene as well as the other nine.

There are several biological reasons which might lead to probes on the same gene giving consistently unrelated readings (alternative splicing, alternative polyadenylation and 3'-5' degradation, come to mind [1,2]). However these do not explain all of the many cases of poor correlation. In [3] we found some technological reasons. In particular, [3] showed that probes containing a large ratio of Guanine (G) to Adenosine (A) bases are likely to perform badly. Subsequently we have found that runs of Gs (which will tend to have a high G/A ratio) also tend to indicate problem probes [4]. This has lead us to ask if there are other *sequences *which might indicate badly behaved probes. We set up an artificial evolutionary system [5,6] to create DNA motifs using a formal computer language grammar [7] to search for DNA sequences which indicate poor probes.

### Grammars and Genetic Programming

Existing research on using grammars to constrain the artificial evolution of programs can be broadly divided in two: "Grammatical Evolution" [8] based largely in Ireland and work in the far east by Whigham [9,10], Wong [11] and McKay [12].

Research in molecular biological computing includes Ross, who induced stochastic regular expressions from a number of grammars to classify proteins from their amino acid sequence [13]. Typically his grammars had eight alternatives. In Stockholm regular expressions have been evolved to search for similarities between proteins, again based on their amino acid sequences [14]. Whilst Brameier in Denmark used amino acids sequences to predict the location of proteins by applying a multi-classifier [15] linear genetic programming based approach [16] (although this can be done without a grammar [17]). A similar technique has also been applied to study microRNAs [18].

## Results and Discussion

**Figure 3:**
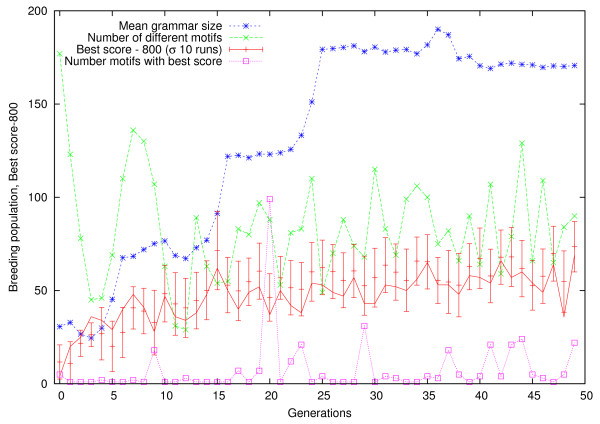
**Evolution of breeding population (best 200 of 1000) of regular expressions to find poor GeneChip probes**. Each generation the positive training cases are replaced leading to fluctuations in the measured best score (solid line). The error bars show the mean and standard deviation of ten GP runs with identical parameters. Note the chosen run is typical and consistently lies within one standard deviation of the mean (+). Diversity remains high and there are usually few motifs with the same highest score (□). In this run the number of distinct motifs (×) (i.e. egrep search strings) is almost identical to the number of distinct grammars. Size is limited (*), apparently by the tree depth limit [17]. However, even so, the system slows down by (≈ ) as evolution proceeds.

**Figure 4:**
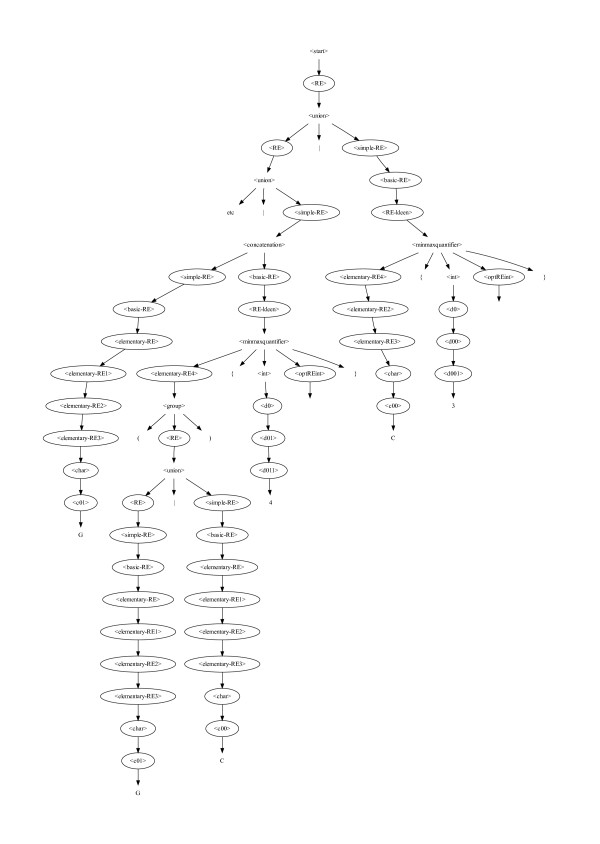
**Right most fragment of grammar of best program in generation 50**. To save space left part is not shown. It would be attached at "etc" (5 arrows from <start>.) Active choice nodes in the BNF (cf. Figure 8) are emphasised by placing them in ovals. The resulting motif is simply the 58 leaf nodes read in left to right order: GC{3}|G{4}|C{4}|CG{1}C{2}|GG{4}C+|G(G|C){4}|G(G|C){4}|C{3}. The fragment just shows the right most end: |G(G|C){4}|C{3}. The motif is equivalent to GGGG|CGCC|G(G|C){4}|CCC.

By the end of the first run (cf. Table 1 and Figure 3) genetic programming (GP) had evolved a probe performance predictor (see Figure 4) equivalent to GGGG|CGCC|G(G|C){4}|CCC. It is obvious that it includes the previous rule (GGGG, [4]) but includes other possibilities. Therefore it finds more poor probes.

**Table 1:** Strongly Typed Grammar GP for GeneChip Correlation Prediction

Primitives:	Possible components of the DNA motif are defined by the BNF grammar (cf. Figure 8).
Performance:	Score = true positives+true negatives, max 1166. (I.e. proportional to the area under the ROC curve or Wilcox statistic [19].) Less large penalty if egrep fails or it matches all probes or none.
Selection:	Each generation the best 200 motifs from the current population of 1000 are used to breed another 1000 motifs.
Initial pop:	Ramped half-and-half 3:7
Parameters:	100% subtree crossover. Max tree depth 17 (no tree size limit)
Termination:	50 generations

**Figure 5:**
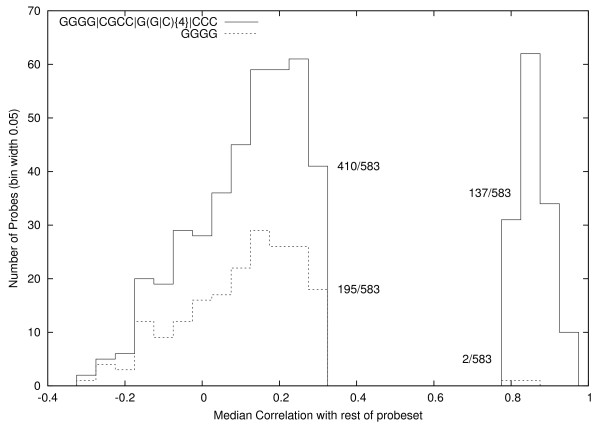
**Performance of evolved motif on its training data versus human generated motif (dashed)**. The solid line shows the new motif finds many more (410 v. 195) poor probes but at the cost of incorrectly identifying 137 good probes as poor.

Inevitably it will also incorrectly predict more high correlation probes as being poor. However its reduced performance on the good probes is more than offset by better performance on the poor probes. See Figure 5. On the last generation, it has a score of 856 (410 true neg + 446 true pos). (GGGG has a score of 776 = 195 + 581.)

**Figure 6:**
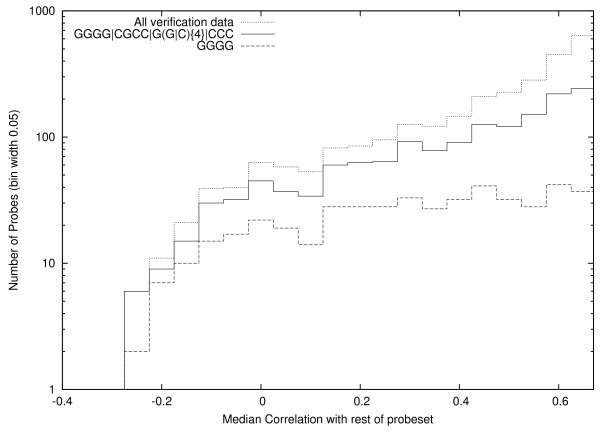
**Performance of evolved and human generated motifs on examples used to check out of sample generalisation**. Again the new motif finds many more poor probes. (Note log scale.)

**Figure 7:**
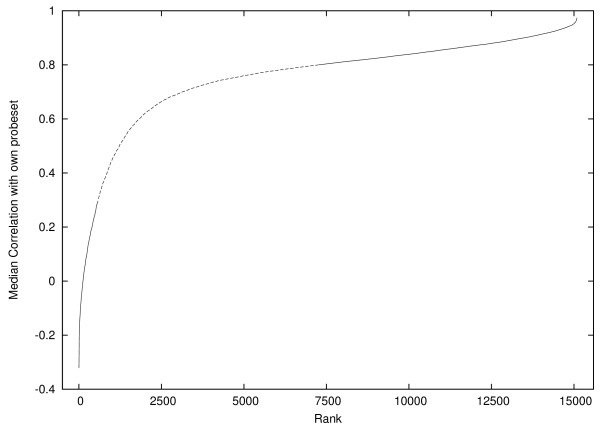
**Training data**. Probes with intermediate values (0.3 ... 0.8) are not used.

The confusion matrix for the evolved regular expression on the whole of the training set (including the 6677 positive middling values which GP never saw) is at the top left of Table 2. As will be described in the methods section, ambiguous middling probes are not used during training, cf. also Figure 7. Nevertheless, to avoid giving an in ated overly optimistic estimate of performance, we present results across the whole range of probe correlations. Whilst its confusion matrix on the verification data is in the middle of Table 2 (The corresponding matrices for GGGG are given in at the bottom of Table 2.) Unlike in many machine learning applications, there is no evidence of over fitting. Indeed the corresponding results for the test set (second matrix of each pair) are not significantly different (χ^2^, 3 dof) from those on the whole training set. The evolved regular expression picks up significantly more (χ^2^, 3 dof) (448 v. 209) poorly performing probes on the test set than the human produced regular expression. Figure 6 shows the number of DNA probes matching the evolved motif against their average correlation with the rest of their probeset.

**Table 2:** Confusion matrices for the evolved motif (top) and original motif (bottom). The performance

GGGG|CGCC|G(G|C){4}|CCC								
Whole training set			Test set			2^*nd *^Test set		

Median Correlation	< 0.3	≥ 0.3		< 0.3	≥ 0.3		< 0.3	≥ 0.3

	410	4448		448	4436		425	4553
-v	173	10061	-v	174	10045	-v	178	9947
								
GGGG								

Whole training set			Test set			2^*nd *^Test set		

Median Correlation	< 0.3	≥ 0.3		< 0.3	≥ 0.3		< 0.3	≥ 0.3

	195	479		209	434		208	462
-v	388	14030	-v	413	14047	-v	395	14038

The performance on the training data is given on the left. "Out of sample" data (i.e. not used for training) gives a better indication of true performance (middle). The number of poor probes correctly predicted is 448 of 622 whist for good probes it is 10 045 of 14 481. The new motif is much better at finding poor probes, 448 v. 209. (Poor probes are those whose average correlation with their own probeset is below 0.3.) But this is at the cost of incorrectly flagging more probes as potentially flawed. Performance does not fall significantly, indicating there is no over fitting. Values for the second (unused) test set are given on the right.

As is common in optimisation [20], almost all the run time is taken by the time to find out the performance score of the motifs. In our case, elapse time is dominated by the command script which runs egrep -c. Typically this takes 8.5 mS per DNA motif. The time taken by gawk to process the BNF grammar, create new grammars, generate the regular expressions, etc., is negligible.

## Discussion

Theoretical and empirical studies of GeneChips confirm that the behaviour of DNA probes tethered to a surface can be quite different from DNA behaviour in bulk solution. This is a new and difficult area and there are not deep pure Physics experimental results. Therefore experimental studies have concentrated on data gathered during normal operation of the chips.

Our automatically generated motif, suggests that in addition to Gs, Cs are important. Indeed the fact that only three consecutive Cs is predictive (whereas four Gs are needed) suggests that Cs are more important than Gs. It is known in GeneChips DNA C-G RNA binds more strongly than DNA G-C RNA [21]. We are tempted to suggest that a CCC sequence on a DNA probe can act as a nucleation site encouraging the probe to bind to GGG on RNA. Indeed the evolved motif suggests that four Gs and mixtures of five Cs and Gs might also form nucleation sites.

The sequence CCC is too short to be specific to a particular gene. GeneChips are designed on the assumption that only RNA sequences which are complementary to the full length of the probe will be stable. However studies have shown that nonspecific targets can be bound to GeneChip probes for several hours even if held only by the nucleation site. This may be why probes with quite short runs of either Cs or Gs can be poorly correlated with others designed to measure the same gene.

## Conclusion

Access to the raw results of thousands of GeneChips (each of which costs several hundreds of pounds) makes new forms of bioinformatic data mining possible.

Millions of correlations between probes in the same probeset, which should be measuring the same gene, show wide variation [22]. Automatically generated regular expressions confirm previous work [3,4] that the DNA sequences from which the probes themselves are formed can indicate poor probe performance. Indeed several new motifs (e.g. CCC) which predict probe quality have been automatically found.

Linux code is available via 

## Methods

### Preparation of Training Data

Previously we had down loaded thousands of experiments from NCBI's GEO [23], normalised them, excluded spatial defects and calculated the correlation between millions of pairs of probes [3,24]. To exclude genes which are never expressed, we selected probesets where ten or more non-overlapping probe pairs had correlations of 0.8 or more. For each probe we use the median value of all 10 of its correlations with other members of its probeset (excluding those it overlaps). This gave 4118 probesets, which were evenly split into three to provide independent training, test and validation data.

Previously we found the "mismatch" probes were often poorly correlated with other measurements for the same gene [3]. Since this is known, we excluded them from this study.

As Figure 7 shows, correlation coefficients cover a wide range. Since we are using correlation only as an indication of how well a probe is working we decided to exclude the middle values from training and instead use probe pairs that were highly correlated (≥ 0.8) or were very poorly correlated (≤ 0.3). Of the 15,092 available training examples, there are 7,832 probes highly correlated with the rest of their probeset but only 583 poorly correlated. To avoid unbalanced training sets, every generation all 583 negative training examples are used and 583 positive examples are randomly chosen from the 7,832 positive examples. Training examples are available via 

### Evolving Regular Expression Motifs

#### BNF grammar of Regular Expression

**Figure 8:**
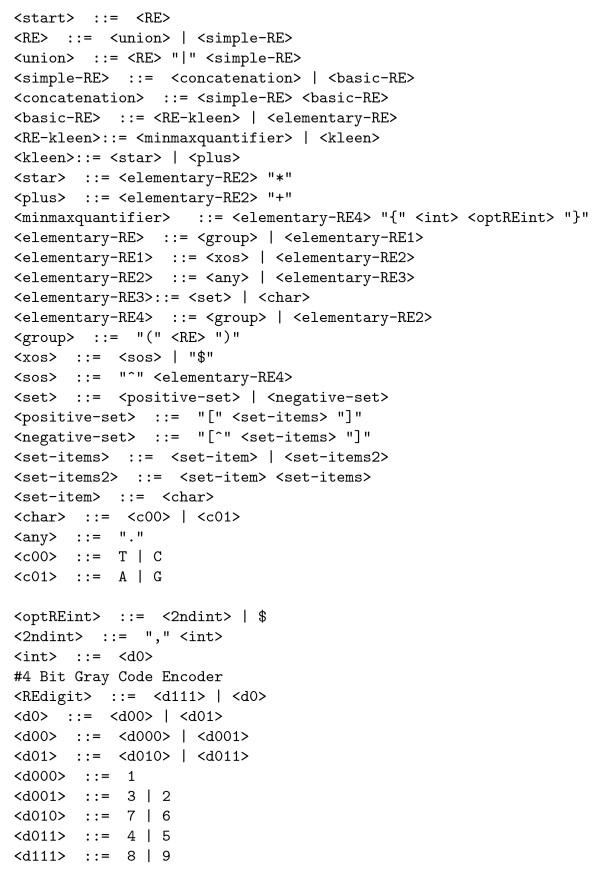
**Grammar used to specify legal regular expressions for use as egrep search strings for testing DNA sequences**.

The BNF grammar used (cf. Figure 8) is an extension of that given by Cameron . In particular, matching the beginning of strings (^) and the {n,m} form of Kleen closure, are also supported. The BNF has been customised for DNA strings. (I.e. <char> need only be A C G and T). Since various combinations of the start of string symbol, null strings and Kleen closure cause egrep to loop, care has been taken to ensure that the new BNF does not permit null strings after ^.

Brameier and Wiuf suggests that the traditional * and + form of Kleen closure are not suitable for bioinformatic applications [18]. Instead they recommend the {n,m} form which explicitly defines both lower (n) and upper (m) limits on the number of times the preceeding symbol must occur. However both {n,m} and traditional Kleen closures are used by evolved solutions. To avoid mutation.awk seeing "Hamming cliffs", the integer quantifiers used in the {n,m} are Gray coded [25]. Similarly the syntax groups together the chemically more similar Pyrimidines (T and C) and Purines (A and G).

Our system supports full positive integer values for the BNF grammar rule minmaxquantifier, however even modest values can lead egrep to hang the computer. Therefore n and m are limited to 1–9. Finally egrep rejects {n,m} if m < n. This is handled by a semantic rule which removes, m from the motif when m is less than n.

### Using the BNF with Genetic Programming

For simplicity, the BNF is written so that grammar rules are either simple substitution rules (e.g. <minmaxquantifier>), rules with exactly two options (e.g. <RE>) or terminals (e.g. "*" and T). In BNF terms, a terminal is a symbol which cannot be substituted in the grammar. Therefore, unlike the BNF rules, it becomes part of the egrep regular expression. The simple substitution rules do not have any element of choice. They, like terminals, cannot be chosen as crossover points or targets for mutation. Their principle use is to enable the rules with options to be kept simple.

**Figure 9:**
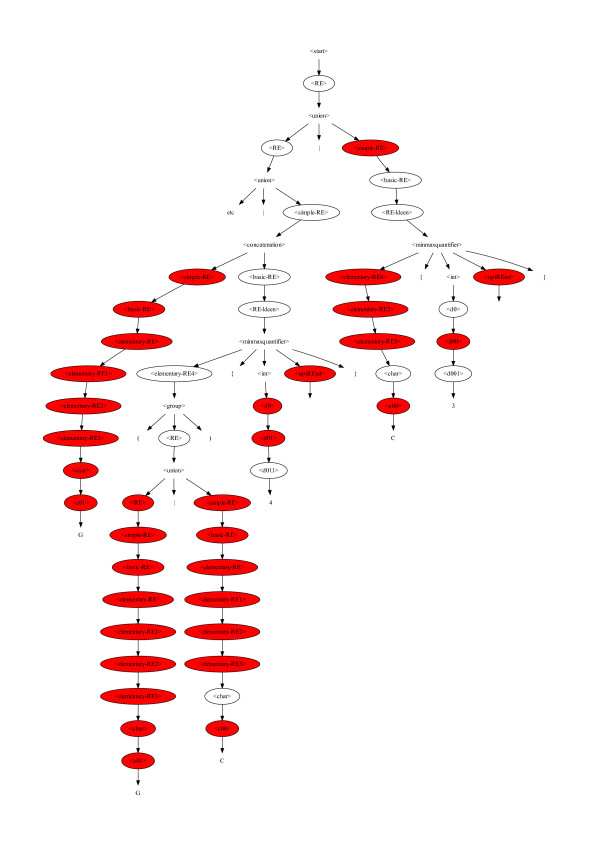
**Fragment of a binary choice tree (ovals) superimposed on grammar (identical to Figure 4)**. Unfilled ovals mean left hand production "0" is to be taken. Shaded ovals indicate right hand production "1" is expanded. Using the BNF grammar shown in Figure 8, the first choice rule following <start> (top) is <RE>. <RE> has two options: <union> and <simple-RE>. This evolved grammar (Figure 4) uses first option (<union>). Hence the first <RE> oval is not filled and the first bit of the equivalent bit string is "0". Thus this tree fragment represents the binary choices: 00 ... 0111111110000111111111111111011101100111010101.

The binary choice rules are the active parts of the syntax. As they are always binary, each egrep regular expression created using the BNF has an equivalent binary string. Each bit in the string corresponds to a BNF rule with two options. The bit indicates which option should be invoked (cf. Figure 9). The BNF grammar is also used to give types to the choices. By using strong typing when creating new motifs from old ones we ensure not only that the new motif is syntatically correct but, since crossover respects the types, they also guide the evolutionary search [26].

#### Creating Random Motifs Using the BNF Grammar

The initial random population is created using ramped half-and-half [27]. It may help to think of this as applying the usual genetic programming ramped half-and-half algorithm to a binary tree (of choice nodes). We start from <start> (at the top of Figure 8) and recursively follow the BNF. However when we reach a rule with options we need to choose one. As in ramped half-and-half we keep track of how deep we are nested. If we have not reached the depth needed to terminate the recursion, we randomly choose one of the options. (As with other strongly typed GPs, if a chosen route through the syntax has no further choices to be made, we may be forced to terminate a recursive branch early.)

To terminate a recursion we choose the "simpler" option. Our BNF has been written so that the simpler option is always on the right. (This is flagged by RE in the rule name.) If there is no "simpler" choice, the choice is made randomly. This mechanism is also used for mutating existing regular expressions.

Although this may seem complex, gawk (Unix' free interpreted pattern scanning and processing language) can handle populations of a million motifs.

#### Creating New Motifs by Mixing BNF Grammars

Creating a new motif from two high scoring motifs is essentially subtree crossover [5] applied to the binary choice tree with the addition of strong type constraints [28]. This is implemented by scanning the grammar used to create the first parent for all the rules with two options. One of these is randomly chosen. For example, suppose the first parent starts <start> <RE> <union> and suppose <union> is chosen as the crossover point. For a grammatically correct child to be produced all that is necessary is that the crossover point chosen in the second parent should also be <union>. (There are complications to do with depth and size limits, which we shall ignore for the time being.) Therefore the second parent is scanned to find all occurrences of <union>. One of them is randomly chosen to be the second crossover point. (If there are none, this crossover is aborted and another initial crossover point is chosen. If we keep failing, eventually another pair of parents is chosen.)

Crossover is based on normal genetic programming (GP) subtree crossover, cf. [[5], Figure 2.5]. The new child is created by copying the start of the first parent, excluding the subtree at the first parent's crossover point. Then genetic material from the subtree at the second parent's crossover point is added. Finally the remainder of the first parent is appended to the child. This is implemented by crossing over the binary choice trees to create a binary choice tree for the new child. Apart from issues of tree size and depth, we are guaranteed that the new binary choice tree will represent a valid DNA motif.

The final step is to recursively trace through the BNF grammar. Each time we come to a rule with two options, we look at the next binary choice. If it is clear, we chose the first option. If it is set, we follow the second option. Each time an BNF terminal is encountered it is appended to the new regular expression. (If the BNF terminal is the null symbol, it is simply ignored.)

#### Evaluating the Performance Score of the DNA Motifs

Each generation, a command file is generated which contains a egrep -c -v '*RE*' command for each motif in the population. (*RE *is the motif i.e. the regular expression.) The command is run on a file holding the DNA sequences of the 583 probes poorly correlated with the rest of their probeset. The same command is also run on a file holding the 583 positive probes selected for use in this generation. The score of the regular expression is based on the difference between the number of lines in the two files which match *RE*. Expressions which either match all probes or fail to match any are penalised by subtracting 583 from their score. See also Table 1. Implementation details can be found in [29].

## Competing interests

The authors are funded by the people of the United Kingdom.

## Authors' contributions

All authors are equally responsible.
